# QT and QTc in Male Patients with Psychotic Disorders Treated with Atypical Neuroleptics

**DOI:** 10.1155/2017/1951628

**Published:** 2017-07-12

**Authors:** Mario Miniati, Marly Simoncini, Federica Vanelli, Caterina Franceschini, Gabriele Massimetti, Claudia Carmassi, Liliana Dell'Osso

**Affiliations:** Section of Psychiatry, Department of Clinical and Experimental Medicine, University of Pisa, Pisa, Italy

## Abstract

**Objective:**

We explored the potential association between antipsychotics and QT/QTc duration changes in hospitalized male patients with psychotic disorders.

**Methods:**

The chart review was conducted on 184 male patients hospitalized between 2013 and 2015 at the Psychiatric Clinic of Pisa, Italy. Patients who were treated with one atypical antipsychotic at the time of the ECG recording were 109/184 (59.2%). QT/QTc were compared considering the atypical antipsychotic received.

**Results:**

96.3% (*n* = 105/109) of the sample showed QTc values ≤ 430 ms; 4 patients (3.7%) had QTc values between 430 and 450 msec (2 with paliperidone, 1 with risperidone, and 1 with olanzapine). The mean QT duration of the overall sample was 368.0 ± 28.0 and the mean QTc 400.1 ± 17.8. QTc values did not reveal statistically significant differences. QT values were significantly different (chi-square = 17.3; df = 5;* p* = .004). Statistically significant differences between aripiprazole and paliperidone (349.0 ± 28.3 versus 390.5 ± 29.8;* p* = .002) and between clozapine and paliperidone (361.1 ± 22.43 versus 390.5 ± 29.8;* p* = .033) were found.

**Conclusions:**

Aripiprazole was the least interfering neuroleptic with QT/QTc. Paliperidone was the atypical neuroleptic with the most relevant difference with aripiprazole, but only on QT.

## 1. Introduction

The prevalence of cardiovascular diseases is high in patients with Psychotic Disorders for a number of reasons, including continued antipsychotic therapy, especially combined therapy with more than one antipsychotic or with other agents [1]. Several physiologic cardiovascular effects are associated with the action mechanism of antipsychotics. For example, the *α*1 adrenergic blockade can raise the risk of syncope or hypotension with an increased incidence of angina episodes [2]. However, one of the most relevant cardiovascular side effects of antipsychotics is QT prolongation [3]. The electrical action of the heart has two components: depolarization, or the stage before the peak of the cardiac wave that leads to the contraction of the heart muscle, and repolarization, the stage in which the heart recharges for the next beat and the heart muscle relaxes. The QT interval is the time during which the system repolarizes. Since this interval depends on heart rate, it is usually measured and reported as the corrected QT interval (QTc). Reference points for normal, borderline, and prolonged QTc are well known [4]. Normal QTc interval is <430 msec for males and <450 msec for females; borderline interval is 431–450 msec for males and 451–470 msec for females; a prolonged QTc interval is considered >450 msec for males and >470 msec for females [4].

If a patient has a borderline QTc, he/she should be assessed at baseline and intermittently after initiation when prescribing any antipsychotic [3]. Moreover, patients whose QTc has increased by 60 msec during antipsychotic treatment would also need to be monitored closely [5].

A number of antipsychotics have led to concern about QT changes. Sertindole was never marketed in the US [6]; mesoridazine was marketed in the US but with a warning regarding dose-related QTc prolongation and associated risk of torsade de pointes [6]. The introduction of ziprasidone was delayed in the US because of concerns about QTc lengthening [7]. Although these drugs are all antipsychotics, they had different side effect profiles, receptor affinities, and mechanisms of action, thus emphasizing the difficulties in making generalizations about them. Moreover, two risk factors unrelated to pharmacotherapy, namely, low potassium levels and inherited long QT syndrome could potentially lead to arrhythmias or torsade de pointes, especially when combined with the cardiovascular effects of antipsychotics [6]. The inherited long QT syndrome is infrequent (1/5000 subjects) and it is due to a genetic defect of cardiac ion channels that might predispose the subject to torsade de pointes [8]. Conversely, hypokalemia could be frequent, because of nausea and vomiting, alcohol abuse, or severe agitation [9].

In this paper, we aimed at examining whether there was an association between antipsychotic use and QT/QTc duration in a common population of hospitalized male patients with Psychotic Disorders, by collecting the ECG during atypical antipsychotic use.

## 2. Methods

### 2.1. Subjects

The chart review was conducted on a total of 184 male patients hospitalized between 2013 and 2015 at the Psychiatric Clinic of the University of Pisa, Italy. Patients who were treated with one atypical antipsychotic were 109/184 (59.2%). Sixty-three patients (63/184; 34.2%) were treated with other drugs, namely, mood stabilizers, benzodiazepines (BDZs), or typical antipsychotics. Twelve patients (12/184; 6.5%) were treated with a second atypical antipsychotic as add-on to the first one.

We collected information from the chart reviews of all patients of whom at least one ECG recording was made during antipsychotic prescription. QT and QTc were compared considering the atypical neuroleptics administered. The chart review was conducted according to the principles expressed in the Declaration of Helsinki [10]. All data were retrieved from the hospital's databases (where data of routine clinical care measures were stored) and analyzed anonymously. No clinical trials or experimental designs were utilized. The institutional medical ethics committee waived the need for an informed consent at the time of hospitalization for the collection of data in the hospital databases both for clinical and for research purposes.

### 2.2. Technical Information

QT and QTc durations were measured by hand and corrected for heart rate using Bazett's formula (QTc). All measurements were analyzed for each patient, based on the best readable recording. We obtained the following information from the hospital records of each patient: age, medical history, status during hospital admission before antipsychotic use (e.g., reason for admission), actual administration and given dose of antipsychotics, and the use of other QT prolonging drugs (Arizona CERT (http://crediblemeds.org/, a reference standard for clinicians)) [6]. ECG was usually collected during the first 24 hours of the hospital admission. If the clinical conditions made the ECG recording impossible (e.g., for a severe psychomotor agitation) the ECG was performed as soon as possible after stabilization. Data of electrolytes measured within 48 hours before or after each ECG were retrieved from charts, using the measurement closest to the moment of recording. Signs of an acute phase response or inflammation at the time of each ECG recording were defined as either body temperature > 39°C, serum CRP level >100 mg/l, or leukocyte count  >11 × 10^9^/L.

### 2.3. Statistics

As QT and QTc were not normally distributed (nonparametric variables), we performed a Kruskal-Wallis Test, followed by a Dunn Test for pairwise post hoc comparisons. Moreover, we compared in two different analyses patients on atypical neuroleptics monotherapy versus patients with a second atypical neuroleptic in add-on and patients with atypical neuroleptics monotherapy versus patients with haloperidol in add-on, with the Mann–Whitney Test. All analyses were performed using SPSS, Version 20.0.

## 3. Results

The ECG of 109 male patients treated with one atypical antipsychotic were collected and compared considering the atypical neuroleptics received, namely, clozapine (*n* = 14), olanzapine (*n* = 13), risperidone (*n* = 12), aripiprazole (*n* = 15), quetiapine (*n* = 43), and paliperidone (*n* = 12).

The mean doses of the atypical neuroleptics were within the standard dosing (clozapine: 232.1 ± 139.8 mg/day; olanzapine: 12.3 ± 6.6 mg/day; paliperidone: 8.5 ± 3.5 mg/day; quetiapine: 285.4 ± 252.0 mg/day; risperidone: 4.2 ± 2.3 mg/day; aripiprazole: 12.8 ± 6.6 mg/day).

The mean age of the selected sample was 42.7 ± 15.0 years. The 96.3% (*n* = 105) of the sample showed QTc values ≤ 430 ms; only 4 patients (3.7%) had QTc values between 430 and 450 msec (2 with paliperidone, 1 with risperidone, and 1 with olanzapine). The characteristics of the selected sample are summarized in Table 1.

No statistically significant differences for age among treatment groups were found. The mean QT duration of the overall sample was 368.0 ± 28.0, and the mean QTc was 400.1 ± 17.8.

Paliperidone was the atypical antipsychotic with the longer mean QT and QTc values (390.5 ± 29.8; 411.4 ± 20.3). As QT and QTc were not normally distributed (nonparametric variables) we performed a Kruskal-Wallis Test, followed by a Dunn Test for pairwise post hoc comparisons. The Kruskal-Wallis Test on QTc values of the overall sample did not reveal statistically significant differences. Conversely, QT values were significantly different (chi-square = 17.3; df = 5; *p* = .004). The Dunn Test for QT pairwise comparisons revealed statistically significant differences between aripiprazole and paliperidone (349.0 ± 28.3 versus 390.5 ± 29.8, resp.; *p* = .002) and between clozapine and paliperidone (361.1 ± 22.43 versus 390.5 ± 29.8, resp.; *p* = .033). QT/QTc comparisons between groups are summarized in Table 2.

Moreover, we found from the chart review that twelve patients (12/184; 6.5%) were treated with a second atypical antipsychotic in add-on: two patients (2/184; 1.0%) were on clozapine plus aripiprazole, two patients on aripiprazole plus olanzapine (2/184; 1.0%), two patients on clozapine plus quetiapine (2/184; 1.0%), two patients on aripiprazole plus quetiapine (2/184; 1.0%), two patients on risperidone plus quetiapine (2/184; 1.0%), one patient on clozapine plus risperidone (1/184; 0.54%), and one patient on clozapine plus paliperidone (1/184; 0.54%). We compared the one hundred and nine patients treated with one atypical antipsychotic with the twelve patients with a second atypical antipsychotic in add-on. No differences were found with the Mann–Whitney Test on QT (QT: 368.0 ± 28.0 versus 362.3 ± 30.2; *p* = .228) and QTc (400.1 ± 17.8 versus 391.6 ± 18.9; *p* = .078), respectively.

A limited number of patients switched from a typical to an atypical antipsychotic or were given exceptionally a typical antipsychotic during hospitalization: one patient received perphenazine (6 mg/day) (1/184; 0.54%), two patients were receiving pimozide (one at 2 mg/day and the second one at 4 mg/day) (2/184; 1.0%), two patients received promazine (50 mg/day and 240 mg/day, resp.) (2/184; 1.0%), and one patient was given zuclopenthixol (60 mg/day) (1/184; 0.54%). A total of eight patients (8/184; 4.3%) were treated with an atypical antipsychotic plus haloperidol as add-on (mean dose: 3.6 ± 1.0; four patients received 3 mg/day; one patient 3.5 mg/day; 2 patients 4 mg/day, and one patient 6 mg/day). Again, no statistically significant differences were found between patients on atypical antipsychotics in monotherapy and those with the add-on of haloperidol, as compared with the Mann–Whitney Test on QT (368.7 ± 28.2 versus 359.0 ± 25.3; *p* = .320) and QTc (400.1 ± 18.3 versus 400.3 ± 10.9; *p* = .991), respectively.

## 4. Discussion

This chart review confirms previous observations on QT/QTc prolongations as occurring infrequently among patients receiving atypical neuroleptics, when well-known risk factors are absent or monitored. Such occurrence can be considered as outliers/random events, and we believe that such random events may better be understood in the context of a naturalistic inpatients setting. Thus, the naturalistic setting might provide clinical information on a wide variety of antipsychotics and not only on a specific drug. Moreover, in agreement with previous observations [11], we believe that an advantage of the naturalistic setting is the consecutive inclusion of a diagnostically heterogeneous and clinically relevant sample with psychotic spectrum symptoms in acute phase. As far as we know, no prospective double-blind studies on QT/QTc have sought to evaluate the outcome within a population of patients with psychotic spectrum disorders treated with atypical neuroleptics. The difficulty of obtaining access to such a population, except during hospitalization, makes that prospective unlikely.

As expected, given the small sample size, we found a limited differential effect between treatment groups. The Kruskal-Wallis Test on QTc values of the overall sample did not reveal statistically significant differences. Only QT values were significantly different. No differences were also found when patients treated with one atypical neuroleptic were compared with patients with a second atypical neuroleptic in add-on, or when haloperidol was administered in add-on.

Our chart review does not allow a “rational” clinical decision making algorithm on the cardiac safe use of a specific atypical neuroleptic. We recommend obtaining at least ECG and potassium on all patients treated with atypical neuroleptics and not only on those with well-known risk factors for TdP, in order to take into account the risk of a QT/QTc prolongation even when considering an infrequent occurrence. Our chart review found that aripiprazole was the least interfering neuroleptic with QT and QTc, confirming previous observations on the minimal risk for QT and QTc prolongation with this drug [1]. Paliperidone, even if with QT and QTc in normal ranges, was the atypical neuroleptic with the most relevant difference with aripiprazole, but again only on QT.

Our chart review had a number of important limitations, such as the small sample size, the absence of a control group, and the evaluation restricted to one ECG per patient, limiting the ability to make inferences on variations of ECG. Moreover, our study did not provide information on the amount of days between ECG collection and the starting of the antipsychotic.

## 5. Concluding Remarks

We believe that naturalistic observations and chart reviews, even if they cannot be viewed as a replacement for controlled clinical trials, might provide a method for gaining insight into treatment choices in a population that is complicated to treat. A better understanding of the relationships between QT/QTc prolongations and antipsychotic administration might help to prevent the occurrence of a cardiac side effect in patients with vulnerability for QT/QTc prolongation. We recommend obtaining at least ECG and potassium in all patients treated with atypical neuroleptics and not only in those with well-known risk factors for TdP. The clinician should always be aware of the potential link between QT/QTc and antipsychotic medications, considering that this link is neither linear nor straightforward.

## Figures and Tables

**Table 1:** Characteristics of the sample (*n* = 109).

	Overall sample (*n* = 109)	Clozapine (*n* = 14)	Olanzapine (*n* = 13)	Risperidone (*n* = 12)	Aripiprazole (*n* = 15)	Quetiapine (*n* = 43)	Paliperidone (*n* = 12)
Age (mean ± SD)	43.0 ± 15.0	39.5 ± 12.8	42.3 ± 14.1	37.3 ± 12.6	38.3 ± 11.6	47.3 ± 16.3	41.3 ± 16.9
Age at onset	24.0 ± 14.1	22.7 ± 13.0	25.7 ± 14.7	22.1 ± 9.4	18.6 ± 4.0	24.4 ± 16.2	31.2 ± 17.3
Illness duration (ys)	18.7 ± 13.3	16.7 ± 10.3	16.5 ± 11.7	14.9 ± 10.6	19.7 ± 11.3	22.4 ± 16.2	13.5 ± 9.4
Body mass index	26.5 ± 4.2	26.3 ± 4.8 (18–37)	25.6 ± 3.3 (22–33)	25.0 ± 2.1 (21–29)	29.5 ± 5.2 (23–40)	26.2 ± 4.2 (19–41)	26.7 ± 2.0 (25–28)
Potassium	4.0 ± 0.4	3.9 ± 0.3 (3.2–4.5)	3.9 ± 0.3 (3.0–4.5)	4.1 ± 0.4 (3.6–5.1)	4.0 ± 0.4 (3.3–5.3)	3.9 ± 0.4 (3.3–5.2)	4.1 ± 0.5 (3.3–5.0)
Sodium	140.9 ± 2.4	140.5 ± 3.5 (133–145)	140.6 ± 2.9 (135–145)	140.6 ± 2.2 (137–145)	141.1 ± 1.1 (139–143)	140.7 ± 2.4 (131–145)	142.5 ± 1.5 (140–145)
Calcium	9.3 ± 0.3	9.4 ± 0.3 (9.0–10.0)	9.1 ± 0.4 (8.1–9.8)	9.4 ± 0.2 (9.1–9.7)	9.5 ± 0.3 (8.9–10.1)	9.3 ± 0.3 (8.1–10.2)	9.3 ± 0.2 (8.8–9.3)
Glycemia	84.3 ± 16.3	86.2 ± 18.5 (55–121)	83.1 ± 15.3 (60–119)	77.5 ± 7.1 (63–87)	86.8 ± 17.1 (67–134)	86.9 ± 19.9 (64–165)	78.6 ± 9.1 (64–93)
Total cholesterol	172.1 ± 40.1	150.5 ± 30.8 (102–223)	178.2 ± 52.4 (123–290)	169.9 ± 28.9 (119–242)	206.8 ± 52.3 (124–295)	162.5 ± 33.2 (88–229)	182.8 ± 29.6 (150–242)
Triglycerides	146.0 ± 79.0	155.7 ± 57.4 (93–259)	141.0 ± 65.5 (47–225)	106.1 ± 22.3 (67–138)	171.7 ± 97.8 (37–339)	150.1 ± 94.5 (56–492)	135.7 ± 57.1 (63–248)
HDL	41.9 ± 13.7	33.2 ± 11.6 (18–53)	43.4 ± 15.1 (27–81)	40.9 ± 7.7 (30–55)	45.6 ± 18.4 (29–93)	42.8 ± 13.6 (22–84)	42.1 ± 12.5 (18–59)
LDL	107.3 ± 36.6	95.4 ± 29.7 (49–151)	106.2 ± 51.1 (50–215)	108.3 ± 24.1 (63–164)	132.1 ± 49.3 (56–235)	97.0 ± 30.9 (38–155)	119.7 ± 24.7 (91–153)
CK	313.1 ± 1160.9	329.6 ± 651.9 (22–2375)	227.5 ± 298.5 (44–942)	201.1 ± 194.4 (60–673)	85.8 ± 57.2 (32–202)	448.9 ± 1813.4 (24–9692)	237.6 ± 160.5 (35–467)
TSH	1.4 ± 1.0	1.4 ± 1.3 (0.2–4.1)	1.2 ± 0.9 (0.2–3.2)	1.5 ± 0.7 (0.4–2.4)	1.2 ± 0.6 (0.2–2.4)	1.4 ± 1.1 (0.1–4.9)	1.2 ± 0.8 (0.4–3.0)

**Table 2:** QT/QTc mean durations in the sample and in treatment groups.

Atypical neuroleptic	QT	QTc
^2^Clozapine (*n* = 14)	361.1 ± 22.4	399.3 ± 11.0
Olanzapine (*n* = 13)	364.5 ± 36.9	393.6 ± 20.5
Risperidone (*n* = 12)	371.8 ± 27.3	403.1 ± 16.6
^1^Aripiprazole (*n* = 15)	349.0 ± 28.3	391.7 ± 23.2
Quetiapine (*n* = 43)	370.6 ± 21.8	401.3 ± 14.8
^1,2^Paliperidone (*n* = 12)	390.5 ± 29.8	411.4 ± 20.3
Overall sample (*n* = 109)	368.0 ± 28.0	400.1 ± 17.8

Kruskal-Wallis Test, followed by a Dunn Test for pairwise post hoc comparisons; ^1^QT paliperidone > QT aripiprazole: *p* = .004; ^2^QT paliperidone > QT clozapine: *p* = .033.
